# GmMYB93 increases aroma formation in soybean by inhibiting the expression of a betaine aldehyde dehydrogenase gene

**DOI:** 10.1007/s42994-025-00236-1

**Published:** 2025-08-08

**Authors:** Jingnan Xu, Faming Lin, Chenhao Zhao, Shaolong Yang, Yu Zhang, Yongchun Shi, Xiaoran Wang, Ran Wang

**Affiliations:** 1 Henan Province Engineering Research Center of Crop Synthetic Biology, College of Life Sciences, Henan Agricultural University, Zhengzhou, 450046 China; 2 State Key Laboratory of Crop Stress Adaptation and Improvement, School of Life Sciences, Henan University, Kaifeng, 475004 China

**Keywords:** 2-Acetyl-1-pyrroline (2-AP), Aroma, Aromatic soybean, *GmBADH2*, Transcriptional repressor

## Abstract

Soybean (*Glycine max*), an exceptionally nutritious crop rich in high-quality proteins and oils, is extensively used in various food products. Aromatic varieties of soybeans are in particular demand. Characterized by its distinctive popcorn-like aroma, 2-acetyl-1-pyrroline (2-AP) is an important volatile compound present in soybeans and other plants. The enzyme betaine aldehyde dehydrogenase (BADH) is closely associated with 2-AP production. However, the transcriptional regulatory network that governs *BADH* gene expression in soybean remains undefined. In this study, we determined that the transcript levels of the *BADH* gene, *GmBADH2*, vary significantly across different soybean organs and differ markedly from those of *GmBADH1*. We showed that GmMYB93 is a transcriptional repressor that directly regulates the expression of *GmBADH2* by binding to the CAGTTA elements in its promoter. Furthermore, the silencing of *GmMYB93* significantly reduced 2-AP accumulation in soybeans. Our findings shed light on the genetic mechanisms underlying soybean aroma formation and lay a foundation for developing novel aromatic soybean varieties.

**Supplementary Information:**

The online version contains supplementary material available at 10.1007/s42994-025-00236-1.

## Introduction

The aromatic compound 2-acetyl-1-pyrroline (2-AP) is indispensable for the quality and delightful “popcorn‐like” fragrance of various crops, including rice (*Oryza sativa*), maize (*Zea mays*), sorghum (*Sorghum bicolor*), and millet (Shan et al. 2015; Wang et al. 2021; Zhang et al. 2022a, 2023). 2-AP is also present in soybean (*Glycine max* [L.] Merr.), which is a major source of vegetable protein in the human diet and an important source of vegetable oil (Jung et al. 2021). Soybean seeds are rich in a diverse array of nutrients and are greatly valued for their versatility in the production of a wide range of food products, such as tofu, soy milk, soy oil, and numerous other soy-based products. Because 2-AP is an important component of soybean seed quality, increasing its content represents an important goal of soybean breeding.

The 2-AP content in the seeds of fragrant soybean varieties ranges from 0.05 to 0.5 mg/kg (Zhang et al. 2021). The biosynthesis of 2-AP is affected by multiple factors in plants. Decreases in *BADH2* and glyceraldehyde-3-phosphate dehydrogenase (*GAPDH*) expression, along with significant increases in triose phosphate isomerase (*TPI*) and Δ1-pyrroline-5-carboxylic acid synthetase (*P5CS*) expression, promote the accumulation of 2-AP (Hinge et al. 2016). The major pathway for 2-AP biosynthesis is thought to rely on the betaine aldehyde dehydrogenase (BADH) pathway (Bradbury et al. 2008; Chen et al. 2008). The knockout of *GmBADH1* and *GmBADH2* markedly increased 2-AP content to 1.59 mg/kg in *gmbadh1 gmbadh2* soybean seeds (Xie et al. 2024). Thus, mutating both *GmBADH1* and *GmBADH2* in non-fragrant soybean varieties through gene editing can generate extraordinarily aromatic soybeans with enhanced taste quality. However, *gmbadh1 gmbadh2* plants have not been thoroughly evaluated, and these plants cannot produce γ-aminobutyric acid (GABA), which is also important for plant growth and development (Li et al. 2021).

GABA, an amino acid with a four-carbon structure, is widely distributed in various organisms (Ahmad and Fariduddin 2024). GABA plays an important role in plant defense responses (Ramesh et al. 2017; Xu et al. 2021). Both the exogenous application of GABA and the upregulation of endogenous GABA levels, through gene editing can promote pesticide metabolism in plants and reduce pesticide contamination during crop production (Shan et al. 2022). BADH also catalyzes the oxidation of betaine aldehyde (BA) to glycine betaine (GB), which enhances plant tolerance to various abiotic stresses, such as drought, salinity, and extreme temperatures, significantly improving plant stress resilience (Chen et al. 2008). Because of the multiple functions of BADH, the regulatory mechanisms for *GmBADH* genes should be explored thoroughly in order to produce soybean lines with both high 2-AP content and sufficient GABA and GB levels.

In this study, with the goal of enhancing seed aroma in soybeans by increasing 2-AP accumulation, we conducted a systematic functional analysis of the promoter region of the soybean *GmBADH2* gene, as this gene is expressed at much higher levels in leaves than *GmBADH1*. We determined that the transcription factor GmMYB93 specifically recognizes and binds to sequences in the *GmBADH2* promoter, significantly inhibiting its transcriptional activity. This inhibition leads to the accumulation of γ-aminobutyraldehyde (GABald) and Δ1-pyrroline, thereby enhancing 2-AP biosynthesis in soybeans. These results elucidate the molecular regulatory mechanism underlying the biosynthesis of the aromatic compound 2-AP in soybean and provide a theoretical foundation and candidate gene resources for the targeted improvement of soybean aroma quality through molecular breeding.

## Results

### Bioinformatic and expression analyses of *GmBADH*

**Fig. 1:**
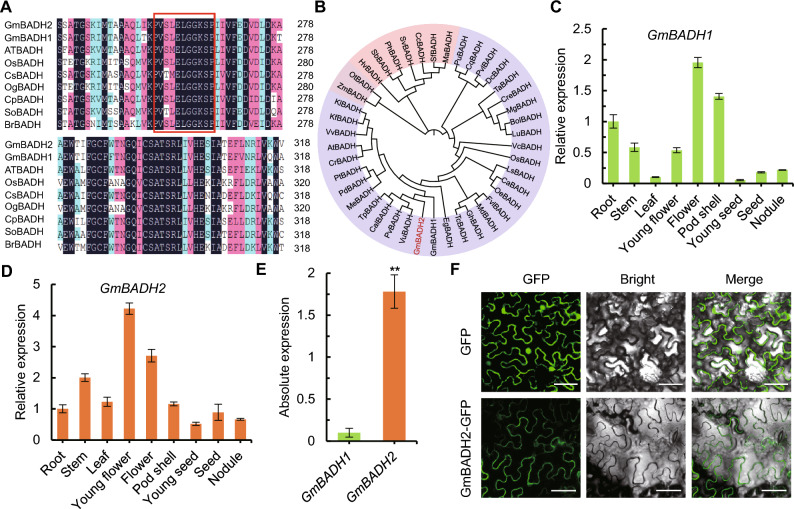
Bioinformatic and expression analyses of *GmBADHs*.** A** The amino acid sequences encoded by the *BADH* coding regions in different plant species. All sequences contain a decapeptide (red frame) that is highly conserved among aldehyde dehydrogenases. The conserved decapeptide sequence in BADH in most plants is VTLELGGKSP, while the decapeptide sequence in BADH in plants such as soybean, sorghum, and maize is VSLELGGKSP. **B** Phylogenetic tree of *BADH* genes. **C** Relative *BADH1* expression levels in different parts of the soybean plant, as determined by RT-qPCR. **D** Relative *BADH2* expression levels in different parts of the soybean plant. **E** Absolute expression levels of *BADH1* and *BADH2* in soybean leaves. **F** Subcellular localization of the mature GmBADH2-GFP protein in transiently transfected *Nicotiana benthamiana* leaves. Bar, 50 µm

We aligned the amino acid sequences encoded by *BADH* genes from various plants. All BADH sequences contained the decapeptide (Val-Thr/Ser-Leu-Glu-Leu-Gly-Gly-Lys-Ser-xPro), a highly conserved motif characteristic of aldehyde dehydrogenases (Fig. 1A). Two homologous *BADH* genes, Glyma.06G186300 and Glyma.05G033500.2, were previously identified in the genome of the soybean variety Williams 82 (Glycine max Wm82.a4.v1) and were named *GmBADH1* and *GmBADH2*, respectively (Juwattanasomran et al. 2012). Here, phylogenetic analysis indicated that *GmBADH1* and *GmBADH2*, which share high similarity with sequences from dicotyledonous plants such as cocoa (*Theobroma cacao*), Arabidopsis (*Arabidopsis thaliana*), and mustard (*Brassica juncea*), are clustered into one branch located far from the *BADH* genes of monocotyledonous plants (Fig. 1B).

To examine the expression patterns of *GmBADH1* and *GmBADH2* in different soybean organs, we performed reverse-transcription quantitative PCR (RT-qPCR). Among the organs examined, mature flowers contained the highest *GmBADH1* and *GmBADH2* mRNA levels, whereas seeds showed the lowest levels (Fig. 1C, D). Notably, the mRNA level of *GmBADH2* in soybean leaves was more than ten times that of *GmBADH1* (Fig. 1E); therefore, we chose *GmBADH2* for further analysis. To investigate the subcellular localization of the encoded protein, we fused the full-length coding sequence of *GmBADH2*, excluding the stop codon, with green fluorescent protein (*GFP*) under the control of the 35S promoter to generate the GmBADH2-GFP fusion construct. This construct was then transiently expressed in the leaves of *Nicotiana benthamiana* seedlings. GFP signals were detected in the plasma membrane, indicating that GmBADH2 localizes to this part of the cell (Fig. 1F).

### Knockout of *GmBADH2* increases the 2-AP content in soybean seeds

**Fig. 2:**
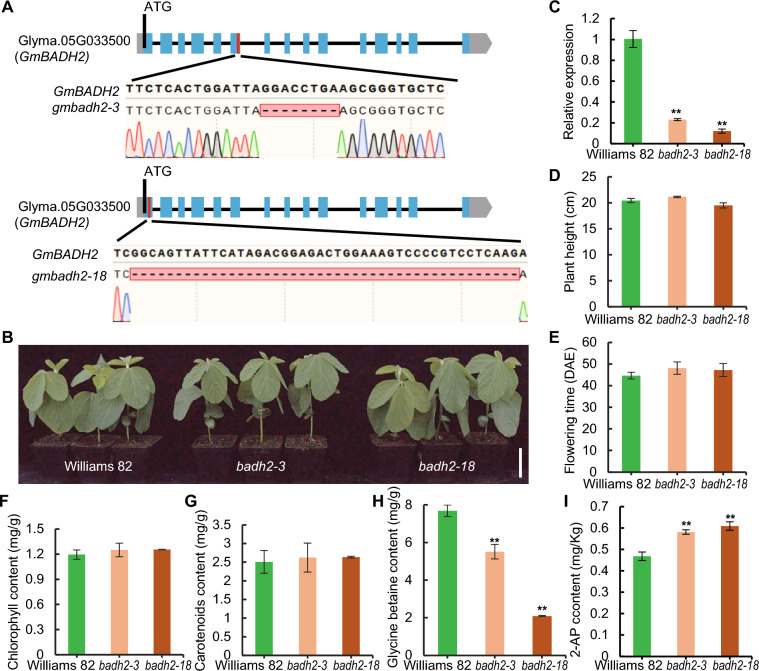
Knockout of *GmBADH2* increases the 2-AP content in soybean seeds.** A** *GmBADH2* crRNA design and mutant genotypes. Exons are represented as black squares and introns as black lines. The three sgRNAs used to target exons 1 and 6 of *GmBADH2* via CRISPR/Cas9 are indicated by red lines. Bar, 500 bp. Gray square, 5′UTR. Blue square, exon. Black line, intron. Gray arrow, 3′UTR.** B** Photograph of Williams 82, *badh2-3*, and *badh2-18* plants at 23 DAE (days after emergence). Bar, 5 cm. **C** Relative *GmBADH2* expression levels in Williams 82, *badh2-3*, and *badh2-18*. **D** Plant height. **E** Flowering time. **F** Chlorophyll content of leaves. **G** Carotenoid content of leaves. **H** Glycine betaine (GB) content of leaves. **I** 2-AP content of dry seeds

To explore the biological function of *GmBADH2* in Williams 82, we used CRISPR/Cas9 gene editing to create *GmBADH2* mutants using three gene editing targets. Following PCR amplification of the target sequences, we subjected the PCR products to DNA sequencing (Fig. 2A). We ultimately obtained two homozygous mutants: *badh2-3*, which has a mutation in the sgRNA2 target; and *badh2-18*, which has mutations in the sgRNA1 target (Fig. 2A). The edited region of *gmbadh2-3* was located between 1194 and 1217 bp of the target coding sequence (from the 5′ to 3′ end), leading to an 8 bp deletion at 1204 bp. The edited region of *gmbadh2-18 * was located between 98 to 152 bp of the target coding sequence, leading to a 44 bp deletion at 102 bp (Fig. 2A). The transcript levels of *GmBADH2* in the *gmbadh2-3* and *gmbadh2-18* mutants were reduced to 22.37% and 9.20%, respectively, of those observed in the wild type (WT), as determined by RT-qPCR analysis (Fig. 2B, C). In summary, we successfully created the *GmBADH2* homozygous mutants *badh2-3* and *badh2-18* through CRISPR/Cas9-mediated genome editing.

We cultivated the mutant plants in an artificial climate incubator with a 16-h light/8-h dark photoperiod at 25 °C  and 50% relative humidity. We observed no obvious differences in the vegetative or reproductive growth of the mutants compared to WT plants (Fig. 2C, D). However, the flowering time of the mutants was delayed by approximately two to three days compared to the WT (Fig. 2E). We also measured the chlorophyll and total carotenoid contents in the mutant leaves and did not find significant differences from the WT (Fig. 2F, G). To further elucidate the biological function of BADH2, we quantified the levels of GB of leaves and 2-AP of dry seeds. The mutation of *BADH2* resulted in the inhibition of GB biosynthesis and a significant increase in 2-AP biosynthesis (Fig. 2H, I).

### GmMYB93 directly binds to the CAGTTA elements in the *GmBADH2* promoter

**Fig. 3:**
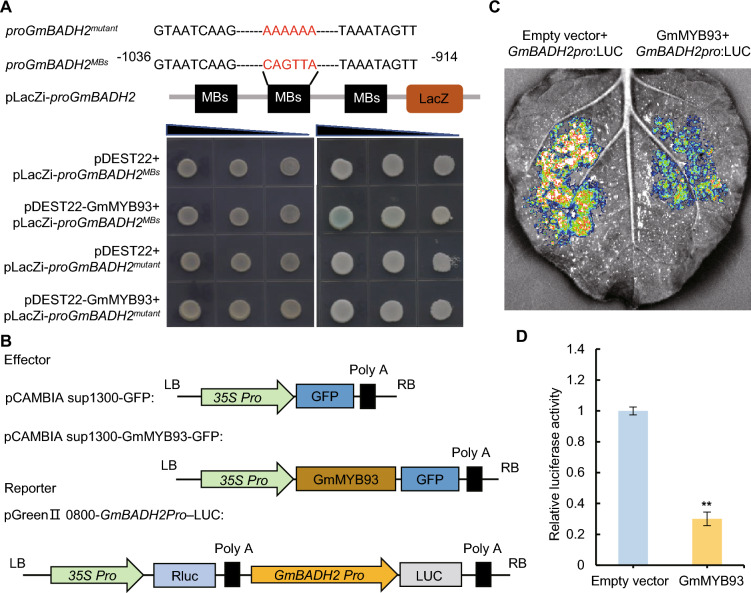
GmMYB93 directly binds to the *GmBADH2* promoter to reduce its expression.** A** Yeast one-hybrid assay showing the binding of GmMYB93 to the indicated promoter fragments. Three copies of the MYB binding domain in the *GmBADH2* promoter were fused upstream of the *LacZ* reporter gene and used for analysis. **B** Diagram of the vectors used for the dual-Luc assay. **C** Transient transformation experiment of tobacco leaves. **D** GmMYB93 directly inhibits the promoter activity of *GmBADH2* in a dual-Luc assay

To elucidate the mechanism regulating *GmBADH2* transcription, we analyzed the 2000 bp sequence upstream of its start codon and identified a CAGTTA motif (a MYB binding element) within the proximal 1 kb of the *GmBADH2* promoter. We generated a construct harboring three copies of the MYB binding element from the *GmBAHD2* promoter fused upstream of the LacZ reporter gene (*GmBADH2*-pro) and used it to perform yeast one-hybrid (Y1H) screening of a yeast monoclonal whole-genome transcription factor library. Yeast cells that were co-transformed with *GmBADH2*-pro and pDEST22-GmMYB93 were able to grow on SC/-Trp-Ura + Gal + Raf + X-gal medium, and the yeast colonies appeared blue, indicating that GmMYB93 binds to the CAGTTA motif in the *GmBADH2* promoter (Fig. 3A).

To further verify that GmMYB93 can bind to the MYB binding element in the *GmBADH2* promoter, we mutated this element to AAAAAA and conducted a Y1H assay with pDEST22-GmMYB93. Upon co-transformation of yeast cells with the construct carrying the mutated promoter sequence and pDEST22-GmMYB93, there was no color change in the yeast colony, indicating that the mutated sequence failed to bind to GmMYB93 (Fig. 3A). Subsequently, we performed a dual-luciferase reporter assay (dual-Luc assay) to delve deeper into the regulatory relationship between GmMYB93 and *GmBADH2*. We inserted a fragment of the *GmBADH2* promoter into the pGreenII 0800-LUC vector to produce the reporter construct and the coding sequence of *GmMYB93* into the pCAMBIA sup1300-GFP vector to form the effector construct (Fig. 3B). As shown in Fig. 3C-D, co-expression of *GmMYB93* and *GmBADH2pro*-LUC resulted in a notable decrease in luminescence intensity.

Given the remarkably high levels of sequence similarity between *GmBADH1* and *GmBADH2*, we conducted an in-depth analysis of the promoter sequence of *GmBADH1* and identified a CAGTTA motif (a MYB binding element) within the 300 bp region of its promoter (Fig. S1A). Subsequently, we constructed a yeast vector for *GmBADH1* using the same method as that used for *GmBADH2* and performed a Y1H assay involving co-transformation with *GmMYB93* to evaluate the interaction between GmMYB93 and the *GmBADH1* promoter. *GmBADH1* and GmMYB93 failed to interact with each other in yeast cells (Fig. S1B). Therefore, GmMYB93 specifically binds to the *GmBADH2* promoter and represses its expression.

### GmMYB93 is a typical member of the MYB transcription factor family

**Fig. 4:**
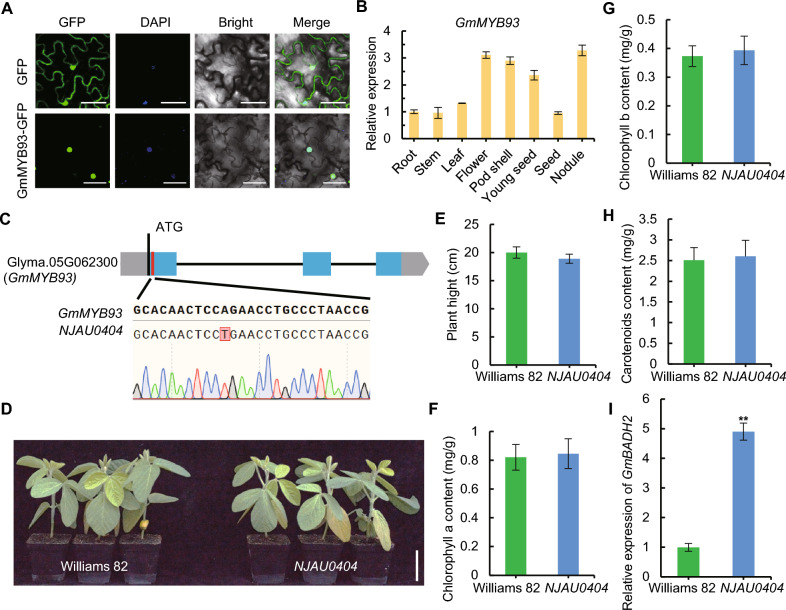
Knockout of *GmMYB93* increases the expression of *GmBADH2* in soybean leaves.** A** Mature GmMYB93-GFP protein localizes to the nuclei of *Nicotiana benthamiana* leaf cells. Bar, 50 µm. **B** Relative *GmMYB93* expression levels in different parts of soybean plants. **C** *GmMYB93* crRNA design and mutant genotypes. Bar, 500 bp. Gray square, 5′UTR. Blue square, exon. Black line, intron. Gray arrow, 3′UTR. **D** Photograph of Williams 82 and *NJAU0404* plants at 22 DAE (days after emergence). Bar, 5 cm. **E** Plant height. **F** Chlorophyll a content of leaves. **G** Chlorophyll b content of leaves.** H** Carotenoid contents of leaves. **I** Relative *GmBADH2* expression levels in the leaves

MYB proteins are classified into four categories based on the number of MYB DNA-binding repeats: the MYB-related, R2R3-MYB, R1R2R3-MYB, and atypical MYB families (Chen et al. 2022). To investigate the classification of GmMYB93, we conducted a multi-species phylogenetic analysis. *GmMYB93* clustered most closely with *AtMYBH*. Within soybean, *GmMYB93* was the most closely related to *GmMYB180*, followed by *GmMYB128*. Amino acid sequence analysis of GmMYB93 revealed a striking similarity to the *Arabidopsis* protein AtMYBH (At5g47390) (Fig. S2A and B). AtMYBH contains four conserved regions: a CCHC-type zinc finger with one region containing an R/KLFGV-type repression domain; a domain with an R1MYB structure that includes a nuclear localization signal, a region containing a putative leucine-rich nuclear export signal (NES), and a region containing an EAR-like repression domain (Huang et al. 2015) (Fig. S2A). Subcellular localization studies in *Nicotiana benthamiana* leaves indicated that the mature GmMYB93 protein exclusively localizes to the nucleus (Fig. 4A), which is consistent with its biological roles and phylogenetic classification. *GmMYB93* expression levels were high in vegetative tissues and gradually decreased during seed development at the ripening stages (Fig. 4B).

To investigate the biological function of GmMYB93 in vivo, we identified a mutant named *NJAU0404*, which harbors a mutation in the GmMYB93 coding region that leads to the premature termination of translation, from an EMS mutant library (Zhang et al. 2022b). Specifically, the coding sequence of *GmMYB93* in *NJAU0404* contains an A-to-T mutation at the 46th bp (from the 5′ to 3′ end) that converts a sequence encoding arginine at the 16th position of the protein into a TGA stop codon (Fig. 4C). There was no significant phenotypic difference between *NJAU0404* and WT plants (Fig. 4D), including in plant height. Similarly, the chlorophyll and carotenoid contents in the leaves of *NJAU0404* showed no obvious differences compared to WT plants (Fig. 4E-H). We also analyzed the expression level of *GmBADH2* in *NJAU0404*. The expression level of *GmBADH2* in the leaves of *NJAU0404* was approximately five-fold higher than that of the WT (Fig. 4I). These results indicate that *GmMYB93* negatively regulates the expression of *GmBADH2* in soybean leaves.

### *GmMYB93* silencing reduces the 2-AP content in soybean seeds

**Fig. 5:**
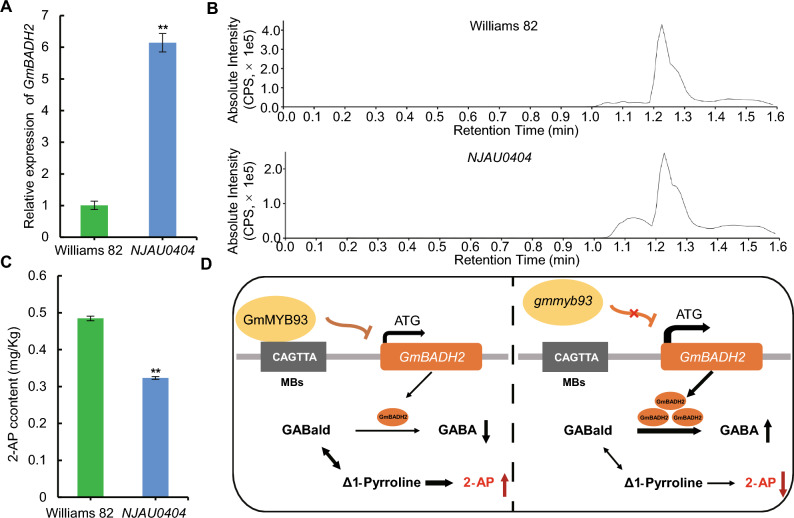
The mutation of *GmMYB93* reduces the 2-AP content in soybean seeds.** A** Relative *GmBADH2* expression level in dry seeds. **B** Peak profile of 2-AP content in seeds of soybean mutant *NJAU0404* and wild-type Williams 82. **C** The 2-AP content of dry seeds. **D** Working model of the role of GmMYB93 in 2-AP biosynthesis. The promoter of *GmBADH2* is bound by the transcription factor GmMYB93, which suppresses its transcription and blocks the conversion of GABald to GABA. This inhibition leads to Δ1-pyrroline accumulation and enhances 2-AP biosynthesis. γ-aminobutyraldehyde (GABald); γ-aminobutyric acid (GABA); 2-acety-1-pyrroline (2-AP). The thickness of the lines represents the intensity of regulation and the reaction rate

Given that *GmBADH2* expression was elevated in the leaves of *NJAU0404,* we reasoned that the expression level of *GmBADH2* and the 2-AP content in seeds might differ between *NJAU0404* and the WT. We measured the expression level of *GmBADH2* in *NJAU0404* and WT seeds via RT-qPCR. *GmBADH2* was expressed at approximately six times higher levels in *NJAU0404* seeds than in the WT (Fig. 5A). Subsequently, we measured the 2-AP content in seeds using UPLC-MS/MS, and found that it was lower in *NJAU0404* seeds than in the WT (Fig. 5B, C). Based on the above findings, we propose the following molecular mechanism for the regulation of 2-AP levels: The promoter region of *GmBADH2* is specifically recognized and bound by the transcription factor GmMYB93. This specific binding inhibits the transcription of *GmBADH2*, thereby preventing the conversion of GABald to GABA. The inhibition of this metabolic pathway promotes the accumulation of Δ1-pyrroline, ultimately leading to increased 2-AP biosynthesis (Fig. 5D).

## Discussion

The 2-AP content varies among different crops and within the same crop at different stages of growth. In commercially available rice, the 2-AP content ranges from 0.032 to 0.552 ppm, with lightly aromatic varieties containing 0.079 ppm of 2-AP (Mathure et al. 2011). The 2-AP content of aromatic soybean seeds ranges from 0.28 to 1.16 ppm (Arikit et al. 2011). Vegetable soybean varieties with value-added traits such as a highly sweet taste and popcorn-like fragrance command premium market prices. One of the major objectives of breeding vegetable soybeans is to develop varieties with high eating quality and enhanced fragrance. Genes associated with several agronomic and quality traits in soybean have been characterized in detail, and functional and structural variations in the form of causal alleles and associated haplotypes have been identified (Kumawat et al. 2016). Natural allelic variation has been detected for *GmBADH2*, a major gene involved in fragrance formation (Juwattanasomran et al. 2011, 2012). However, although much is known about the genetic basis of 2-AP biosynthesis and the accumulation of its aromatic metabolites, few studies have focused on the transcriptional regulation of enzymes that influence the formation of aroma compounds in soybean.

In this study, we created aromatic soybean lines by inactivating the soybean *GmBADH2* gene through genome editing, consistent with previous findings using *gmbadh1 gmbadh2* plants (Xie et al. 2024). Our results demonstrate that decreasing *GmBADH2* expression is a viable approach for increasing 2-AP levels in soybean seeds.

The expression pattern of *GmBADH2* uncovered in this study suggest that the expression of *GmBADH2* is finely regulated by transcription factors. The expression patterns of *GmBAHD2* and *GmMYB93* are similar, and both exhibit rhythmicity, suggesting a potential correlation between these genes. Our findings suggest that MYB93 suppresses the expression of *GmBAHD2*, which contrasts with the positive correlation observed between their expression profiles. These observations point to the existence of additional protein components that may influence the regulation of *GmBAHD2* by MYB93. Members of the MYB transcription factor family are characterized by the presence of a MYB domain. This domain, approximately 51–52 amino acids long, comprises a series of highly conserved amino acid residues and spacer sequences (Stracke et al. 2001). A common feature of MYB transcription factors is the presence of a conserved MYB DNA-binding domain, which is composed of 1–4 incomplete repeat motifs (R). Each repeat sequence comprises three α-helices, each containing approximately 50–53 amino acid residues. The second and third α-helices form a helix-turn-helix (HTH) structure, allowing MYB transcription factors to bind to the major groove of their target DNA sequence, thereby regulating target gene expression (Chen et al. 2022; Wu et al. 2024). *Arabidopsis* *AtMYB93* encodes a negative regulator of lateral root development that is induced by auxin, as *atmyb93* mutants are insensitive to auxin specifically with respect to lateral root development (Gibbs et al. 2014). *MdMYB93*, an apple (*Malus domestica*) ortholog of *AtMYB93*, regulates the biosynthesis and organization of suberin (Legay et al. 2016). AtMYBH regulates hypocotyl elongation in *Arabidopsis* in response to darkness (Kwon et al. 2013). AtMYBH also acts as a transcriptional repressor, playing crucial roles in regulating plant development and dark-induced leaf senescence (Huang et al. 2015). Therefore, it is of significant value to further investigate the multifaceted functions of *MYB93* in soybean.

The transcription factor OsWRKY19 was recently shown to enhance fragrance by negatively regulating *OsBADH2* expression in rice. Interestingly, *OsWRKY19* also enhances agricultural traits in rice plants (Li et al. 2024). Thus, identifying the transcriptional activators and repressors that modulate *GmBADH* gene expression represents a promising yet largely unexplored avenue for quality improvement. Our findings demonstrate that the newly identified transcription factor GmMYB93 increases 2-AP content by negatively regulating *GmBADH2* expression, providing an important genetic resource for further research on aroma improvement in soybean.

## Materials and methods

### Plant materials and growth conditions

The soybean *GmBADH2* mutants (*gmbadh2-3*, *gmbadh2-18*) and wild-type (WT) Williams 82 plants were used in this study. The plants were grown in an artificial climate incubator with a 16-h light/8-h dark photoperiod, temperature of 25 °C, light intensity of 250 μmol m^−2^ s^−1^, and relative humidity of 50%. Samples for gene expression analysis were obtained from WT soybean plants.

### Bioinformatic analysis of *GmBADH2* and *GmMYB93*

The genomic data and annotation information for the common soybean variety Williams 82 were downloaded from Phytozome (https://phytozome.net/). The protein sequence, genomic sequence, and coding sequence of *Glycine max BADH2* (*GmBADH2*) were obtained through a BLAST comparison at the National Center for Biotechnology Information (https://www.ncbi.nlm.nih.gov/) using the Arabidopsis *BADH2* (*AT3G48170*) coding sequence as a query. Other plants in the NCBI non-redundant protein sequence database were searched using the BLASTP program (https://blast.ncbi.nlm.nih.gov/Blast.cgi). Homology matching of the above sequences was performed using DNAMAN, and evolutionary trees were constructed using the neighbor-joining method with MEGA11 software. The cis-acting elements in the 2000 bp upstream promoter region of *GmBADH2* were predicted using PlantCARE (https://bioinformatics.psb.ugent.be/webtools/plantcare/html/).

### Subcellular localization of GmBADH2 and GmMYB93

The open reading frame of *GmBADH2* was cloned and inserted into the pCAMBIA sup1300 vector, which was subsequently used to express GmBADH2 fused with GFP at its C-terminus under the control of the cauliflower mosaic virus 35S promoter. The pCAMBIA and sup1300-GmBADH2-GFP recombinant vectors were introduced into *Agrobacterium tumefaciens* strain GV3101 by electroporation. The positive strain was selected on Luria–Bertani (LB) medium supplemented with 35 µg/mL of kanamycin. This strain was used to infect *Nicotiana benthamiana* leaves for the transient expression of GmBADH2-GFP. Fluorescence signals were detected using an A1R HD25 laser scanning confocal microscopy system (Japan Nikon). Similar methods were used for the subcellular localization of GmMYB93. Primers are listed in Supplementary Table S1.

### Construction of gene editing vectors and identification of homozygous *GmBADH2* mutants

The appropriate target site (protospacer-adjacent motif, PAM) of *GmBADH2* was selected using CRISPR Multi Targeted (http://www.multicrispr.net/index.html). The vector and T0 transgenic plants were provided by Wuhan Boyuan Biotechnology Co., Ltd. Mutation sites in gene edited-positive plants were identified by PCR using mutation site-specific primers. After obtaining homozygous *GmBADH2* mutants, the seeds of T2-positive transgenic plants were collected for subsequent experiments. Primers are listed in Supplementary Table S1.

### Generation of a standard curve for 2-AP

A stock solution of 2-AP was prepared with an initial concentration of 100 µg/mL. The stock solution was diluted using hexane to create a series of standard solutions with specific concentrations of 5, 10, 15, 20, and 25 µg/mL. A micro-injector was used to dispense 1 µL of each standard solution into the analytical instrument (Qsight LX 50 coupled with Qsight 420 triple quadrupole mass spectrometer [PerkinElmer, USA]), with three replicates per concentration to ensure data reliability. A standard calibration curve was constructed based on the relationship between the peak area of 2-AP and its corresponding injected concentrations.

### Extraction and measurement of pigment components

Fresh soybean leaves were cut into pieces and mixed well. A 0.1 g leaf sample was placed into a 10 mL centrifuge tube, with each sample divided into three portions as technical replicates. For pigment extraction, 5 mL of extraction buffer (ethanol/acetone/water = 4.5:4.5:1) was added to each tube, and the sample was incubated in the dark at 4 °C for ~ 12 h until the leaves turned completely white. Using the extraction buffer as a blank control, the absorbance of the chlorophyll extract was measured at wavelengths of 645 nm and 663 nm using a UV–Vis spectrophotometer, while the absorbance of the carotenoid extract was measured at a wavelength of 450 nm.

### RNA extraction and RT-qPCR

Total RNA was extracted from soybean tissues using a FastPure Plant Total RNA Isolation Kit (Vazyme, Nanjing, China) according to the manufacturer’s instructions. First-strand cDNA was synthesized using HiScript III RT SuperMix with gDNA Wiper (Vazyme, Nanjing, China) as per the manufacturer’s protocol. Primers for RT-qPCR were designed using DNA sequences from the Phytozome 13 database. Taq Pro Universal SYBR qPCR Master Mix was used for quantitative PCR amplification using an ABI StepOnePlus Real-Time PCR System. The thermal cycling conditions were as follows: initial denaturation at 95 °C for 30 s, followed by 40 cycles of 95 °C for 10 s and 60 °C for 30 s. Each experiment was conducted with three biological replicates. Primers are listed in Supplementary Table S1.

### Yeast one-hybrid assay

The promoter fragments of *GmBADH2* were cloned and fused into the pLacZi vector. The full-length *GmMYB93* sequence was inserted into the pDEST22 vector, resulting in pDEST22-GmMYB93, with the empty pDEST22 vector serving as a negative control. pDEST22-GmMYB93 was then introduced into yeast strain EYG48, which was co-transformed with these reconstructed vectors. The transformants were inoculated on SD/-Trp-Ura medium (without tryptophan and uracil), incubated for 3 days at 28 °C, and then transferred to SC/-Trp-Ura + Gal + Raf + X-gal medium (without tryptophan and uracil, with the addition of raffinose, galactose, and X-gal). Primers are listed in Supplementary Table S1.

### Dual-luciferase reporter (LUC) assay

A dual-luciferase assay was performed to validate the binding of GmMYB93 to the *GmBADH2* promoter. To create the reporter construct, the *GmBADH2* promoter fragment was inserted into the pGreenII 0800-LUC vector, while the coding sequence of *GmMYB93* was inserted into the pCAMBIA sup1300-GFP vector to form the effector construct. These constructs were introduced into the Agrobacterium strain GV3101, and a mixture of *A. tumefaciens* carrying the reporter or effector constructs was infiltrated into *Nicotiana benthamiana* leaves. The infiltrated leaves were incubated in the dark for 24 h, followed by 24 h of light exposure. Promoter activity was quantified by calculating the ratio of firefly luciferase (LUC) enzyme activity to the internal reference Renilla luciferase (REN) using a multifunctional microplate reader. The LUC/REN ratio was calculated in the absence of GmMYB93. Primers are listed in Supplementary Table S1.

### Statistical analysis

The significant differences between the two compared samples were analyzed using Student’s *t*-test. Statistical significance was set at *P* < 0.05.

## Supplementary Information

Below is the link to the electronic supplementary material.

Supplementary file1 (DOCX 992 KB)

Supplementary file2 (XLSX 11 KB)

## Data Availability

All data supporting the findings of this study are available in this paper and supplementary information.
