# Supplementary material for: GmMYB93 increases aroma formation in soybean by inhibiting the expression of a betaine aldehyde dehydrogenase gene
Source: aBIOTECH. 2025 Aug 8;6(3):569–79. doi: 10.1007/s42994-025-00236-1 (PMC12454755; doi:10.1007/s42994-025-00236-1)
Supplement: Supplementary file 1 — Supplementary file1 (DOCX 992 KB) [file 42994_2025_236_MOESM1_ESM.docx]

**GmMYB93 increases aroma formation in soybean by inhibiting the expression of a betaine aldehyde dehydrogenase gene**

Jingnan Xu^1#^, Faming Lin^1,2#^, Chenhao Zhao^1^, Shaolong Yang^1^, Yu Zhang^1^, Yongchun Shi^1^, Xiaoran Wang^1*^, Ran Wang^1^^*^

^1^ Henan Province Engineering Research Center of Crop Synthetic Biology, College of Life Sciences, Henan Agricultural University, Zhengzhou 450046, China

^2^ State Key Laboratory of Crop Stress Adaptation and Improvement, School of Life Sciences, Henan University, Kaifeng 475004, China

^＃^These authors contributed equally to this article.

***Correspondence:**

Ran Wang

[wangran@henau.edu.cn](mailto:wangran@henau.edu.cn)

Xiaoran Wang

xiaoranwang@henau.edu.cn

**Supplementary Materials：**


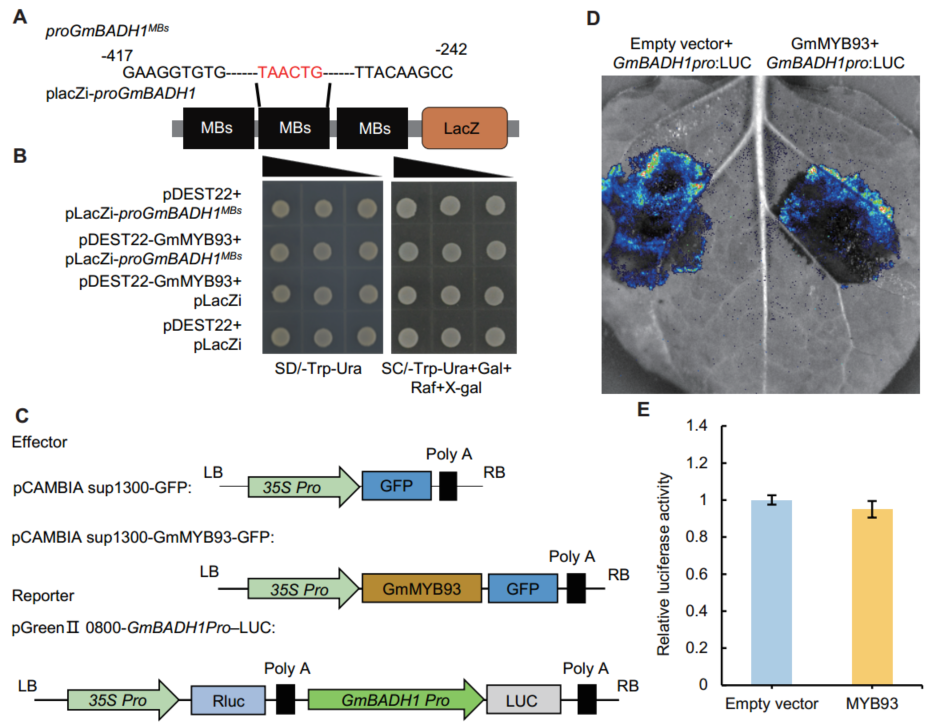


**Fig. S1** GmMYB93 protein can’t directly binds to the promoter region of *GmBADH1*. **A** The MBs motif of the *GmBADH1* Promoter was repeated three times and fused upstream of the LacZ reporter gene. **B** Yeast-one-hybrid experiment of GmMYB93 and *GmBADH1*. **C** Schematic of vectors used for the dual-Luc assay. **D-E** GmMYB93 directly inhibits the promoter activities of *GmBADH1* in a dual-Luc assay.


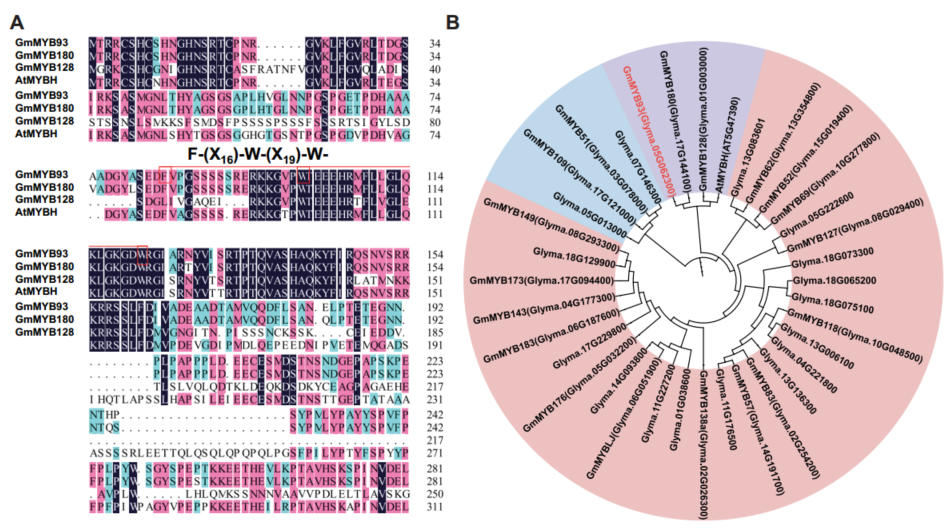


**Fig. S2** Bioinformatics analysis of GmMYB93. **A** The amino acid sequence alignment of GmMYB93. **B** Phylogenetic tree of GmMYB93.


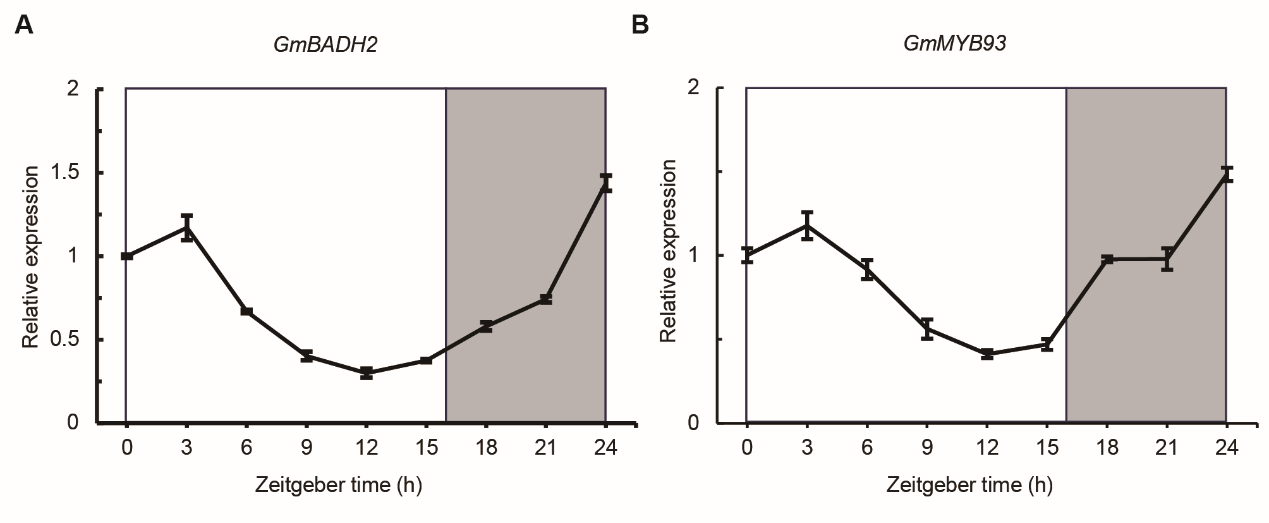


**Fig. S3** The analysis of the diurnal expression patterns of *GmBADH2* and *GmMYB93*. **A** The expression trend of *Gm**BADH2* within 24 hours, the white area represents 16 hours of light exposure and the gray area represents darkness. **B** The expression trend of *GmMYB93* within 24 hours, the white area represents 16 hours of light exposure and the gray area represents darkness.
